# Residual Disease in Patients with Axial Spondyloarthritis: A Post-Hoc Analysis of the QUASAR Study †

**DOI:** 10.3390/jcm11123553

**Published:** 2022-06-20

**Authors:** Salvatore D’Angelo, Carlo Salvarani, Francesca Marando, Giuliana Gualberti, Lucia Novelli, Giacomo Curradi, Giovanni Tripepi, Annalisa Pitino, Roberta Ramonda, Antonio Marchesoni

**Affiliations:** 1 Rheumatology Department of Lucania, San Carlo Hospital of Potenza, 85100 Potenza, Italy; 2 Rheumatology Unit, Azienda USL-IRCCS di Reggio Emilia and Università di Modena and Reggio Emilia, 42123 Reggio Emilia, Italy; carlo.salvarani@ausl.re.it; 3 AbbVie Srl, 00185 Rome, Italy; francesca.marando@abbvie.com (F.M.); giuliana.gualberti@abbvie.com (G.G.); lucia.novelli@abbvie.com (L.N.); giacomo.curradi@abbvie.com (G.C.); 4 National Research Council—Institute of Clinical Physiology (CNR-IFC), 89124 Reggio Calabria, Italy; gtripepi@ifc.cnr.it; 5 Institute of Clinical Physiology (IFC-CNR), 00185 Rome, Italy; pitino@ifc.cnr.it; 6 Rheumatology Unit, Department of Medicine-DIMED, University of Padova, 35131 Padova, Italy; roberta.ramonda@unipd.it; 7 Rheumatology, Humanitas San Pio X, 20122 Milan, Italy; marchesoni@tiscali.it

**Keywords:** ASDAS, ASQoL, ankylosing spondylitis, BASDAI, EQ-5D-5L, HRQoL, residual disease, remission, low disease activity, pain/discomfort, tiredness/fatigue

## Abstract

In this study, we evaluated the presence of residual disease in patients with axial spondyloarthritis (axSpA) in remission/low disease activity (LDA) status. This cross-sectional post-hoc analysis of the QUASAR study involving 23 rheumatology centres across Italy included adults with axSpA classified according to the Assessment of SpondyloArthritis International Society criteria. Patients with inactive disease (score < 1.3) or at least LDA status (score < 2.1) at baseline visit according to Ankylosing Spondylitis Disease Activity Score were investigated to evaluate how residual disease activity impacts patients’ quality of life. They were assessed using the Ankylosing Spondylitis Quality of Life (ASQoL) and EuroQoL 5-Dimension 5-Level (EQ-5D-5L) questionnaires. This study included 480 patients with axSpA (mean age, 47.5 ± 12.9 years, 64% male). In total, 123 patients (25.6%) had inactive disease and 262 (54.6%) had at least LDA. Using the ASQoL, ranges of 10–25% and 20–40% of patients with inactive disease and with LDA status, respectively, experienced tiredness/fatigue. Despite being classified with inactive disease, 48.8% of patients reported light pain/discomfort according to the EQ-5D-5L, with 4.1% reporting moderate pain/discomfort, whereas 55.7% of patients with LDA reported light pain/discomfort and 13% had moderate pain/discomfort. Using the ASQoL questionnaire, in patients with at least LDA, a higher proportion of women compared with males and a higher proportion of patients > 48 years of age (vs. patients ≤ 48 years) experienced tiredness. In this post-hoc analysis, ≥25% of axSpA patients in remission/LDA status were still burdened by residual disease, mainly characterised by pain and fatigue.

## 1. Introduction

Axial spondyloarthritis (axSpA) is a chronic inflammatory disease characterised by inflammation and new bone formation in the sacroiliac joints and spine and include non-radiographic axSpA and radiographic axSpA (also known as ankylosing spondylitis (AS)) [1,2,3]. Treatment options for axSpA include non-steroidal anti-inflammatory drugs and biologic disease-modifying anti-rheumatic drugs (bDMARDs) that target tumour necrosis factor-alpha (TNFα) or interleukin (IL)-23/17 axes [4,5,6,7,8]. The efficacy of TNFα and IL-23/17 inhibitors in controlling signs and symptoms of axSpA has been shown in clinical trials [4,5,6]. However, as also confirmed by observational studies, a proportion of patients treated with biologic drugs do not achieve low disease activity (LDA) or remission status.

In addition, despite being in clinical remission or LDA states, a significant number of patients with other forms of SpA, such as psoriatic arthritis (PsA), can still suffer from a burden of residual disease that significantly impacts their health-related quality of life (HRQoL) [9,10,11]. Data from randomised controlled trials are of limited use in quantifying residual disease burden because they have inherent biases, such as external validity, unrealistic treatment decisions, and a non-representative patient population. To overcome these drawbacks, an increasing number of studies have relied on data from real-world studies, which provide a more accurate measure of the burden of residual disease.

The most up-to-date tool used for the assessment of disease activity in axSpA is the Ankylosing Spondylitis Disease Activity Score (ASDAS), which defines LDA or inactive disease status scores of <2.1 and <1.3, respectively [12,13]. To date, few studies are available that have explored the burden of residual disease in axSpA patients with inactive disease or who have achieved LDA status, as per the ASDAS definition [14]. To address this unmet need, the aim of this post-hoc analysis of the Italian multicentre QUASAR study was to evaluate the presence of residual disease in patients with axSpA who had reached remission/low disease activity LDA status.

## 2. Patients and Methods

### 2.1. Patient Population

Patients included in the QUASAR study [15,16] were aged ≥18 years and classified with axSpA according to the Assessment of SpondyloArthritis International Society axSpA criteria [1] and capable of understanding and completing the questionnaires. Exclusion criteria were the participation in a clinical study for the treatment of axSpA or a life expectancy of ≤1 year. Patients could, therefore, have been previously or currently treated. In this post-hoc analysis of the QUASAR baseline visit data, only patients with an ASDAS score value recorded at baseline were considered. This analysis was specifically undertaken to evaluate the presence of residual disease in patients with axSpA who had inactive disease defined as an ASDAS score of <1.3 or presented with at least LDA status (i.e., ASDAS score of <2.1) at the baseline visit. Patients categorised as having at least LDA status also comprised patients with inactive disease (i.e., in remission). The QUASAR study was approved by each institutional ethics committee/review board (23 approvals; first approval obtained on 18 February 2014 by the Ethics Committee of ASL Lecce, Italy; n. 7; no further approval number foreseen by local regulations due to the epidemiological nature of the study) and all patients provided written informed consent in accordance with existing applicable laws (DL 196/03). The study was conducted in accordance with the Declaration of Helsinki.

### 2.2. Disease Activity Measures

Assessment measures used to collect data in the QUASAR study have previously been described in detail [15,16]. At each outpatient visit, disease activity was assessed using the ASDAS, Bath Ankylosing Spondylitis Disease Activity Index (BASDAI) [17], Physician’s Global Assessment of Disease Activity on a 0 to 100 visual analogue scale (PhGA-VAS). The BASDAI consists of six questions relating to the five major symptoms of AS: fatigue/tiredness, spinal pain, joint pain or swelling, areas of localised tenderness, and morning stiffness duration and severity on a scale from 0–10. The presence of axSpA symptoms (inflammatory spinal pain and stiffness) were also recorded in the patients’ case report forms. This post-hoc analysis was based on the baseline visit data.

### 2.3. HRQoL Questionnaires

The Ankylosing Spondylitis Quality of Life (ASQoL) [18] and EuroQoL 5-Dimension 5-Level (EQ-5D-5L) questionnaires were used to assess HRQoL [19]. The ASQoL questionnaire measures the impact of axSpA on HRQoL from the patient perspective and includes 18 items [18]. Fatigue/tiredness are symptoms frequently seen in patients with inflammatory rheumatic diseases and can significantly impact their QoL [14,20]. Therefore, in this post-hoc analysis, we focused on three specific questions (questions 7, 8, and 12) related to tiredness/fatigue. The EQ-5D-5L questionnaire is a generic tool measuring HRQoL and comprises two parts: a descriptive system and a VAS [19]. The descriptive component includes five single-item dimensions (mobility, self-care, usual activities, pain/discomfort, and anxiety/depression) to describe the health status of the patient. In this analysis, we considered Question 4 related to pain/discomfort. The VAS evaluates general health on a continuous response scale ranging from 0 (worst possible health state) to 100 (best possible health state). All questionnaires used to assess HRQoL were previously validated in the Italian language [21,22,23].

### 2.4. Evaluation of the Burden of Residual Disease

Patients were identified as presenting with residual disease upon confirmation of the presence of fatigue/tiredness or pain/discomfort as assessed by AsQoL or EQ-5D-5L or based on individual clinical judgement of the referring physician, despite their ASDAS status indicating remission or LDA. To explore the impact of residual disease in terms of fatigue, we specifically examined three items from the ASQoL: items 7 (“I feel tired all day”), 8 (“I have to stop what I am doing to rest”), and 12 (“I get tired easily”) for tiredness/fatigue. Pain and discomfort were assessed using the EQ-5D-5L, item 4 (“pain and discomfort” on a 5-point scale). Analysis of other residual symptoms in patients in remission or LDA but classified as having “signs and symptoms” was also undertaken (collected by the physician or reported by the patient) to better characterise the specific clinical picture.

### 2.5. Statistical Analysis

The sample size determination of the original QUASAR study (512 patients) has been described in detail elsewhere [15]. In this post-hoc analysis, only patients having an ASDAS score value recorded at baseline (*n* = 480) were considered. Quantitative variables were described using mean, standard deviation, median, and interquartile range (IQR) as appropriate, and qualitative variables through absolute and relative frequencies. Baseline characteristics were compared (between inactive and LDA subgroups) using the Fisher exact test or Mann–Whitney test. Frequency distribution analyses by group in remission and LDA categories were also performed for the BASDAI, PhGA, EQ-5D-5L, and ASQoL. We also calculated two measures of asymmetry/tailedness of the dataset distribution represented by skewness and kurtosis. A symmetrical dataset is represented by a skewness equal to 0. If the kurtosis is greater than 3, then the dataset has heavier tails than a normal distribution. If the kurtosis is less than 3, then the dataset has lighter tails than a normal distribution. Subanalyses of patients by ASDAS score for age (≤48 years vs. >48 years) and disease duration (≤7 years vs. >7 years) were based on the median value. Statistical analyses were performed using Stata software version 13 (StataCorp, College Station, TX, USA). A *p*-value of <0.05 was considered statistically significant.

## 3. Results

### 3.1. Baseline Clinical Characteristics

The QUASAR study included 512 patients with axSpA who were consecutively enrolled across 23 Italian centres from May 2014 to April 2015. The baseline and clinical characteristics have previously been described in detail [15,16]. A summary of demographic and baseline clinical characteristics of the 480 patients included in this post-hoc analysis is presented in Table 1. The majority of patients were male (*n* = 307, 64%) and the mean age was 47.5 ± 12.9 years. Approximately half of patients (*n* = 218, 45.4%) had extra-muscular manifestations (EMMs) of axSpA (psoriasis, uveitis, and inflammatory bowel disease), and a similar percentage of patients (*n* = 206, 42.9%) had comorbidities, the most frequent being hypertension (*n* = 77, 16%), allergy (*n* = 29, 6%), and anxiety/depression (*n* = 20, 4%). The majority of patients (*n* = 423; 82.6%) were currently receiving anti-TNF biologic treatment (median duration of 36.4 months) and showed mild disease activity as indicated by low median C-reactive protein (CRP) levels (3.4 mg/L; IQR 1.5–7 mg/L) and low median BASDAI (2.74; 1.1–5.0) and ASDAS (1.9; 1.3–2.9) values.

### 3.2. Characteristics of Patients in Remission (ASDAS < 1.3) or LDA (ASDAS < 2.1)

Among patients with inactive disease and those with LDA (Table 1), 123 patients (25.6%) were in complete remission and 262 (54.6%) had an LDA status. Patients in remission were younger, had longer disease duration, lower CRP-levels, and lower burden of comorbid diseases, and in the non-remission group, most were males. Interestingly, a significantly greater proportion of patients in remission had EMMs compared with patients not in remission. In patients with LDA status, a similar pattern emerged compared with patients with higher levels of disease activity (ASDAS ≥ 2.1).

### 3.3. Residual Disease, Including Pain and Fatigue, in Patients Achieving Inactive Disease and LDA

**Figure 1:**
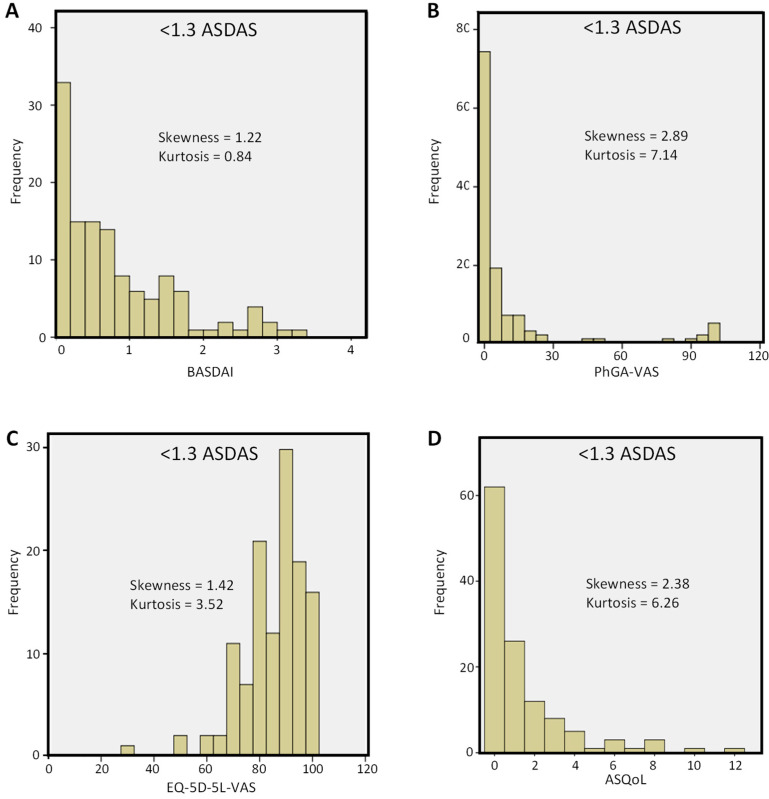
Frequency distribution of (**A**) BASDAI, (**B**) PhGA-VAS, (**C**) EQ-5D-5L-VAS, and (**D**) ASQoL scores in patients with axSpA in remission (ASDAS < 1.3). ASDAS = Ankylosing Spondylitis Disease Activity Score; ASQoL = Ankylosing Spondylitis Quality of Life; BASDAI = Bath Ankylosing Spondylitis Disease Activity Index; EQ-5D-5L-VAS = EuroQoL 5-Dimension 5-Level visual analogue scale component of EQ-5D-5L to describe the health status of the patient; PhGA-VAS = Physician Global Assessment-visual analogue scale.

**Figure 2:**
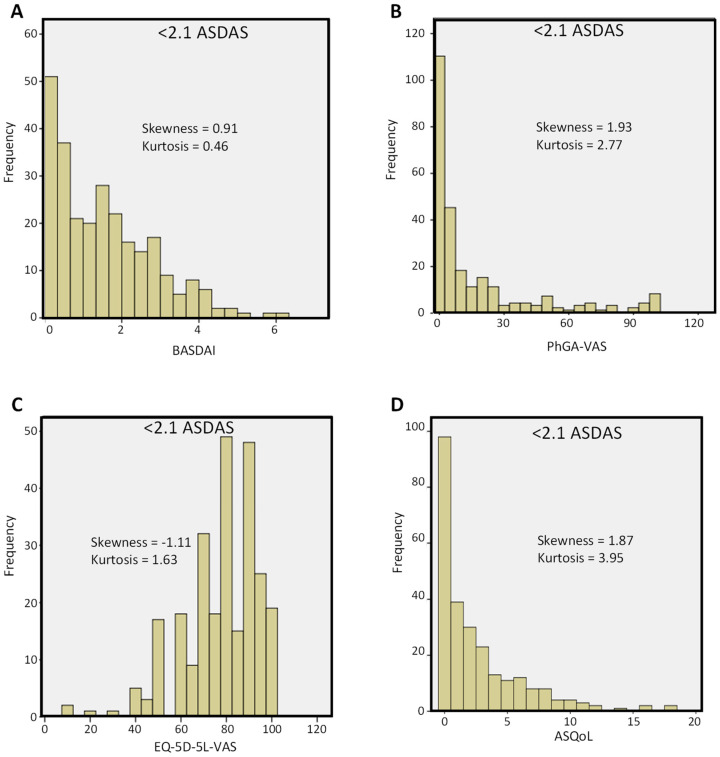
Frequency distribution of (**A**) BASDAI, (**B**) PhGA-VAS, (**C**) EQ-5D-5L-VAS, and (**D**) ASQoL scores in patients with axSpA in at least low disease activity state (ASDAS < 2.1). ASDAS = Ankylosing Spondylitis Disease Activity Score; ASQoL = Ankylosing Spondylitis Quality of Life; BASDAI = Bath Ankylosing Spondylitis Disease Activity Index; EQ-5D-5L-VAS = EuroQoL 5-Dimension 5-Level visual analogue scale component of EQ-5D-5L to describe the health status of the patient; PhGA-VAS = Physician Global Assessment-visual analogue scale.

The frequency distributions of disease activity (BASDAI and PhGA-VAS) and HRQoL measures (EQ-5D-5L-VAS and ASQoL) were skewed towards values indicating good disease control (Figure 1 and Figure 2). Mean PhGA values were 11.1 ± 25.3 and 16.9 ± 26.5, respectively, for patients in remission and those who had LDA. Nevertheless, a distinct proportion of patients showed values consistent with residual disease, especially those with LDA. 

Median scores for the six questions relating to BASDAI are shown in Table 2. In patients in remission, the highest scores were observed for fatigue: question 1 (“How would you describe the overall level of fatigue/tiredness you have experienced?”), question 2, related to overall level of pain in the neck, back, or hip (“How would you describe the overall level of AS neck, back, or hip pain you have had?”), and question 5, related to the level of morning stiffness (“How would you describe the level of morning stiffness you have had from the time you wake up?”). As expected, scores for these same questions were higher in patients with LDA.

Approximately half (48.8%) of patients in remission experienced slight pain or discomfort as measured using the EQ-5D-5L (Table 3). In patients with LDA, 78 (29.8%) had no pain/discomfort; however, 146 (55.7%) had slight pain/discomfort and 35 (13.4%) had moderate pain/discomfort. Considering the ASQoL questionnaire, we observed that despite being classified as being in remission by the ASDAS score, some patients still reported feeling tired all day (question 7, 9.8%) or needing to stop what they were doing to rest (question 8, 8.9%). Additionally, nearly one-quarter of patients stated that they get tired easily (question 12, 24.4%) (Table 3). The percentages for questions 7 and 8 were more than two-fold in patients with LDA status (approximately 20% each), and 100 patients (38.2%) stated that they get tired easily (question 12).

### 3.4. Signs and Symptoms in Patients Achieving Inactive Disease and LDA

A summary of signs and disease symptoms are summarised in Table 4. Nearly half of patients (*n* = 56, 45.5%) experienced symptoms although they were in remission. Furthermore, residual pain (for any location) and stiffness were still present in 31 patients (25.2%) and 40 (32.5%), respectively. Patients in LDA state reported a higher frequency of disease symptoms (*n* = 161, 61.5%), with pain (*n* = 112, 42.7%) and stiffness (*n* = 113, 43.1%) being the predominant symptoms reported.

### 3.5. Impact of Demographic and Other Features on Residual Disease

The impact of demographic and clinical characteristics, such as sex, age, and disease duration, on questions related to tiredness from the ASQoL and pain/discomfort from the EQ-5D-5L questionnaires was also explored. Using the ASQoL questionnaire, more women than men reported feeling tired all day (question 7) or having to stop what they were doing to rest (question 8), although this difference did not attain statistical significance (Table 5). However, in the LDA group, a significantly higher percentage of women compared with males reported feeling tired all day (question 7, 35.4% vs. 14.8%; *p* < 0.001), having to stop what they were doing to rest (question 8, 30.4% vs. 14.8%; *p* = 0.006), and getting tired easily (question 12, 46.8% vs. 34.4%; *p* = 0.05) (Table 5). 

With regard to age, no difference was noted using the cut-off value of 48 years, with the exception of question 8 (I had to stop what I was doing to rest) in the LDA group, which encompassed a significantly higher proportion of patients >48 years of age than patients ≤48 years (25.7% vs. 15.0%; *p* = 0.04) (Table 6). 

Using the ASQoL questionnaire, no differences emerged when groups were stratified by disease duration (≤7 years or >7 years) in patients in remission or in LDA (Table 7). No significant differences were observed for sex, age, or disease duration using the EQ-5D-5L questionnaire in patients in remission or LDA status.

## 4. Discussion

This multicentre survey reflects the real-life status quo of the current routine care and management of PsO patients in Italy.

The QUASAR study showed that that only one-quarter (25.6%) of patients with axSpA had inactive disease (ASDAS < 1.3) and just over half of patients (54.6%) were in LDA status (ASDAS < 2.1). Considering that more than 80% of patients were receiving anti-TNF biologic treatment, these results seem to point towards a clear unmet need in the management of these patients.

The ASDAS is currently considered the best instrument to assess disease activity in axSpA [12,13,24]. However, in the treat-to-target strategy for the management of SpAs [25], even if clinical remission/inactive disease of musculoskeletal and EMMs is the target to be achieved, no specific tool to measure this target is currently available. Optimal disease control is a wide concept that implies the absence of symptoms, such as pain and fatigue, and good control of EMMs and comorbidities, in addition to clinical remission/inactive disease [26]. The results of this post-hoc analysis of the QUASAR study suggest that despite being in clinical remission or LDA, patients with axSpA may still have a relevant burden of residual disease. Among patients in clinical remission, approximately 50% reported mild pain/discomfort and about 4% reported moderate pain/discomfort according to the EQ-5D-5L. The same figures in the LDA groups were about 56% and 13%, respectively. In addition to pain, stiffness was often reported (33% and 43%, respectively, for patients in remission and with LDA). Indeed, it would be expected that patients who are on bDMARDs would be more severe and have a higher burden of comorbidities, which can in turn influence the disease perception by the patient.

Fatigue/tiredness are symptoms frequently seen in patients with inflammatory rheumatic diseases and can significantly impact their QoL [20]. In this study, 10–25% of patients in remission and 20–40% of those with LDA experienced tiredness/fatigue according to the ASQoL.

The higher frequency of EMMs in patients in remission (56.1%) and with LDA (49.1%) remains unclear. It is possible that patients with EMMs underwent a more aggressive therapy with bDMARDs that resulted in better control of the rheumatic disorder. The association between the presence of EMMs and fatigue/tiredness is recognised [9,14,27], although we did not observe any obvious association. Further analysis involving a larger sample size in axSpA patients over time are needed to verify the role of EMMs in this context. Among sex, age, and disease duration, only female sex showed a significant association, with a greater burden of residual disease in the LDA subgroup. These results are in contrast with previous observations where, among other variables, younger age, shorter disease duration, and LDA at baseline were associated with ASDAS remission [28].

To our knowledge, only one other study to date has investigated the burden of residual disease in a population of patients with axSpA [14]. The 87 of 262 patients (33%) who achieved ASDAS-ESR LDA showed residual disease burden in musculoskeletal manifestations, as well as in pain, fatigue, PhGA scores, and mental health. Information on treatment (i.e., biologic therapy) was not described in this study. Overall, our results are in line with the findings from this study.

Several studies have addressed the issue of residual disease in patients with PsA, another condition belonging to the SpA group [9,10,11,29]. All of those studies showed that patients with good disease control still had a relevant burden of disease. In an observational study, it was observed that just under half (49%) of patients with PsA in minimal disease activity (MDA) had active skin disease [29]. In a study performed in the Netherlands involving 292 patients newly diagnosed with PsA [30], 8% achieved MDA and 74% achieved LDA according to the Disease Activity Index for Psoriatic Arthritis tool after 1 year of usual care. Of these patients, 38% reported a global assessment score of >2.0 and 43% had a pain score of >1.5. Similarly, a study performed in Turkey in patients with PsA showed residual disease in joint count, enthesitis, and dactylitis, and 22.2% and 11% of patients in MDA had symptoms of depression and anxiety, respectively, in addition to an elevated level of fatigue [9]. 

In the Canadian Biologic Treatment Registry Across Canada (BioTRAC) prospective study, 223 patients with rheumatoid arthritis, AS, or PsA were treated for 12 months with the biologics infliximab, golimumab, or ustekinumab [11]. MDA was achieved in 44.8% of patients at 12 months, and the most commonly unmet MDA criteria were patient-reported pain (25%), patient global assessment (15%), and Psoriasis Area Severity Index (12%).

## 5. Study Limitations

Because this was a cross-sectional study in a population of patients with axSpA, data on long-term changes in residual symptoms (and their causative factors) were not collected. However, QoL and disease activity measures were shown to improve up to 1 year in our follow-up analysis. Given that most patients were receiving biologic therapy, they cannot be considered representative of real-life clinical practice, where a substantial number of patients are not on bDMARDS. Some characteristics of the patients were not fully investigated, and the possible impact of structural damage and other diseases, such as osteoarthritis, fibromyalgia, or anxious-depressive syndrome, could not be established.

## 6. Conclusions

In this cross-sectional post-hoc analysis of the QUASAR study, about 25% of patients were in inactive disease and 54.6% had LDA. A relevant proportion of these patients showed residual disease, especially under the form of pain/discomfort and tiredness/fatigue. These findings underscore the importance of close monitoring of laboratory and clinical features of patients with axSpA treated with anti-TNF therapy, even when they are in clinical remission or LDA according to ASDAS. Our data identify two unmet needs: (1) The use of the ASDAS tool may not be “comprehensive” of the global disease burden in patients with axSpA and (2) anti-TNF therapy (80% of our patients were treated with these drugs) may not “cover” all the domains, in particular those that refer to pain and fatigue. It is important to stress that residual disease recognised by physicians frequently will not result in a change in the therapeutic management of the patient [31]. In this context, current tools used to identify patients still burdened with residual disease are limited. Specific domains that more closely reflect underlying inflammatory states are needed [32]. Further studies would be helpful to understand how optimising therapeutic approaches in these patients may lead to reduced residual disease and associated symptoms.

## Figures and Tables

**Table 1:** Demographics and baseline clinical characteristics of all evaluable patients stratified by the ASDAS cut-off values.

Variable	All Patients	<1.3 ASDAS	≥1.3 ASDAS	*p*-Value	<2.1 ASDAS	≥2.1 ASDAS	*p*-Value
	*n* = 480	*n* = 123 (25.6%)	*n* = 357 (74.4%)		*n* = 262 (54.6%)	*n* = 218 (45.4%)	
Age, mean ± SD	47.5 ± 12.9	45.1 ± 12.3	48.3 ± 12.9	0.014	45.97 ± 12.7	49.2 ± 12.9	0.006
Male	307 (64)	91 (74)	216 (60.5)	0.007	183 (69.8)	124 (56.9)	0.003
Marital status							
Single	108 (22.5)	35 (28.5)	73 (20.4)		67 (25.6)	41 (18.8)	
Married/living with partner	340 (70.8)	81 (65.9)	259 (72.5)		181 (69.1)	159 (72.9)	
Widow/bachelor	7 (1.5)	1 (0.8)	6 (1.7)	0.3	3 (1.1)	4 (1.8)	0.24
Separated/divorced	25 (5.2)	6 (4.9)	19 (5.3)		11 (4.2)	14 (6.4)	
Smoking status							
Never smoker	243 (50.6)	63 (51.2)	180 (50.4)		129 (49.2)	114 (52.3)	
Current smoker	120 (25)	26 (21.1)	94 (26.3)	0.42	62 (23.7)	58 (26.6)	0.30
Previous smoker	117 (24.4)	34 (27.6)	83 (23.2)		71 (27.1)	46 (21.1)	
Disease duration, median (IQR)	7 (2–12)	9 (4–14)	6 (2–12)	<0.001	8 (3–12)	6 (1–12)	<0.001
HLA-B27							
Positive	244 (50.8)	80 (65.0)	164 (45.9)		148 (56.5)	96 (44.0)	
Negative	156 (32.5)	26 (21.1)	130 (36.4)	0.001	71 (27.1)	85 (39.0)	0.012
Not performed	80 (16.7)	17 (13.8)	63 (17.6)		43 (16.4)	37 (17.0)	
CRP (mg/L), median (IQR)	3.4 (1.5–7)	1.5 (0.7–3)	4.9 (2.2–8.5)	<0.001	2.45 (1–5)	5.3 (3–12.6)	<0.001
ASDAS, median (IQR)	1.9 (1.3–2.9)	0.8 (0.6–1.1)	2.4 (1.8–3.2)	<0.001	1.3 (0.8–1.7)	3.0 (2.6–3.5)	<0.001
ASQoL, median (IQR)	4 (1–10)	0 (0–2)	7 (3–12)	<0.001	1 (0–4)	10 (6–13)	<0.001
BASDAI, median (IQR)	2.7 (1.1–5.0)	0.6 (0.2–1.2)	3.9 (2.2–5.8)	<0.001	1.4 (0.5–2.4)	5.3 (3.8–6.8)	<0.001
PhGA-VAS, median (IQR)	12.0 (2.0–48.2)	2.0 (0.0–7.0)	21.0 (5.0–54.0)	<0.001	4.0 (0.0–21.0)	31.0 (9.0–63.0)	<0.001
EQ-5D-5L-VAS, median (IQR)	70.0 (50.0–85.0)	90 (80–95)	60 (50–80)	<0.001	80 (70–90)	50 (40–70)	<0.001
Extra-muscular manifestations of axSpA	218 (45.4)	69 (56.1)	149 (41.7)	0.006	130 (49.6)	88 (40.4)	0.04
Concomitant disease	206 (42.9)	43 (35)	163 (45.7)	0.04	95 (36.3)	111 (50.9)	0.001
Biologic treatment	423 (82.6)	123 (91.9)	278 (77.9)	0.001	222 (84.7)	169 (77.5)	0.043

ASDAS = Ankylosing Spondylitis Disease Activity Score; axSpA = axial spondyloarthritis; ASQoL = Ankylosing Spondylitis Quality of Life; BASDAI = Bath Ankylosing Spondylitis Disease Activity Index; CRP = C-reactive protein; EQ-5D-5L-VAS = EuroQoL 5-Dimension 5-Level visual analogue scale component of EQ-5D-5L to describe the health status of the patient; IQR = interquartile range; PhGA-VAS = Physician Global Assessment—visual analogue scale. Data are presented as *n* (%) unless noted otherwise.

**Table 2:** Patient responses to specific questions related to BASDAI by ASDAS.

Question	<1.3 ASDAS *n* = 123	<2.1 ASDAS *n* = 262	≥2.1 ASDAS *n* = 218
Q1 (fatigue)	0.7 (0.1–2.0)	1.6 (0.4–3.4)	6.1 (4.6–7.6)
Q2 (pain)	0.4 (0.1–1.2)	1.2 (0.3–3.0)	6.5 (4.7–8.0)
Q3 (pain)	0.1 (0–0.6)	0.4 (0–1.4)	4.5 (2.3–6.9)
Q4 (discomfort)	0.2 (0–0.6)	0.4 (0–1.8)	4.7 (1.9–6.7)
Q5 (stiffness)	0.4 (0–1.2)	1.0 (0.1–2.5)	6.0 (3.4–7.7)
Q6 (stiffness)	0.2 (0–1.1)	0.7 (0–2.1)	4.5 (2.1–6.2)

ASDAS = Ankylosing Spondylitis Disease Activity Score; BASDAI = Bath Ankylosing Spondylitis Disease Activity Index. Data are presented as median (interquartile range). Q1. How would you describe the overall level of fatigue/tiredness you have experienced? Q2. How would you describe the overall level of AS neck, back, or hip pain you have had? Q3. How would you describe the overall level of pain/swelling in joints other than neck, back, hips you have had? Q4. How would you describe the level of discomfort you have had from an area tender to touch or pressure? Q5. How would you describe the level of morning stiffness you have had from the time you wake up? Q6. How long does your morning stiffness last from the time you wake up?

**Table 3:** Quality of life in patients with axial spondyloarthritis using ASQoL and EQ-5D-5L stratified by ASDAS.

Variable	<1.3 ASDAS	<2.1 ASDAS	≥2.1 ASDAS
	*n* = 123	*n* = 262	*n* = 218
ASQoL			
Q7: I feel tired all day	12 (9.8%)	55 (21.0%)	136 (62.4%)
Q8: I have to stop what I am doing to rest	11 (8.9%)	51 (19.5%)	149 (68.3%)
Q12: I get tired easily	30 (24.4%)	100 (38.2%)	168 (77.1%)
EQ-5D-5L (Q4: pain and discomfort)			
1. I have no pain or discomfort	58 (47.2%)	78 (29.8%)	8 (3.7%)
2. I have slight pain or discomfort	60 (48.8%)	146 (55.7%)	52 (23.9%)
3. I have moderate pain or discomfort	5 (4.1%)	35 (13.4%)	117 (53.7%)
4. I have severe pain or discomfort	0 (0)	3 (1.1%)	37 (17%)
5. I have extreme pain or discomfort	0 (0)	0 (0)	4 (1.8%)

ASDAS = Ankylosing Spondylitis Disease Activity Score; ASQoL = Ankylosing Spondylitis Quality of Life; EQ-5D-5L = EuroQoL 5-Dimension 5-Level. Data are presented as *n* (%).

**Table 4:** Residual signs and symptoms in patients with axial spondyloarthritis stratified by ASDAS.

Sign or Symptom	<1.3 ASDAS	<2.1 ASDAS	≥2.1 ASDAS
	*n* = 123	*n* = 262	*n* = 218
Any symptom	56 (45.5%)	161 (61.5%)	195 (89.4%)
Pain (any location)	31 (25.2%)	112 (42.7%)	174 (79.8%)
Lower back pain	19 (15.4%)	74 (28.2%)	135 (61.9%)
Upper back pain	3 (2.4%)	18 (6.9%)	47 (21.6%)
Neck pain	8 (6.5%)	36 (13.7%)	66 (30.3%)
Joint pain	11 (8.9%)	46 (17.6%)	93 (42.7%)
Stiffness	40 (32.5%)	113 (43.1%)	160 (73.4%)
Arthritis	1 (0.8%)	6 (2.3%)	36 (16.5%)
Dactylitis	0 (0.0%)	2 (0.8%)	13 (6%)
Enthesitis	6 (4.9%)	22 (8.4%)	45 (20.6%)

ASDAS = Ankylosing Spondylitis Disease Activity Score. Data are presented as *n* (%).

**Table 5:** Quality of life in patients with axial spondyloarthritis using ASQoL and EQ-5D-5L stratified by sex and ASDAS.

Variable	Male	Female		Male	Female	
	<1.3 ASDAS	<1.3 ASDAS	*p*-Value	<2.1 ASDAS	<2.1 ASDAS	*p*-Value
ASQoL	*n* = 91	*n* = 32		*n* = 183	*n* = 79	
Q7: I feel tired all day	7 (7.7%)	5 (15.6%)	0.3	27 (14.8%)	28 (35.4%)	<0.001
Q8: I have to stop what I am doing to rest	6 (6.6%)	5 (15.6%)	0.15	27 (14.8%)	24 (30.4%)	0.006
Q12: I get tired easily	22 (24.2%)	8 (25%)	1	63 (34.4%)	37 (46.8%)	0.05
EQ-5D-5L						
1. I have no pain or discomfort	47 (51.6%)	11 (34.4%)		59 (32.2%)	19 (24.1%)	
2. I have slight pain or discomfort	41 (45.1%)	19 (59.4%)	0.1	102 (55.7%)	44 (55.7%)	0.24
3. I have moderate pain or discomfort	3 (3.3%)	2 (6.3%)		20 (10.9%)	15 (19.0%)	
4. I have severe pain or discomfort	0 (0%)	0 (0%)		2 (1.1%)	1 (1.3%)	

ASDAS = Ankylosing Spondylitis Disease Activity Score; ASQoL = Ankylosing Spondylitis Quality; EQ-5D-5L = EuroQoL 5-Dimension 5-Level. Data are presented as *n* (%).

**Table 6:** Quality of life in patients with axial spondyloarthritis using ASQoL and EQ-5D-5L stratified by age and ASDAS.

Variable	Age ≤ 48 Years	Age > 48 Years		Age ≤ 48 Years	Age > 48 Years	
	<1.3 ASDAS	<1.3 ASDAS	*p*-Value	<2.1 ASDAS	<2.1 ASDAS	*p*-Value
ASQoL	*n* = 77	*n* = 46		*n* = 153	*n* = 109	
Q7: I feel tired all day	7 (9.1)	5 (10.9)	0.76	32 (20.9)	23 (21.1)	1
Q8: I have to stop what I am doing to rest	6 (7.8)	5 (10.9)	0.75	23 (15.0)	28 (25.7)	0.04
Q12: I get tired easily	19 (24.7)	11 (23.9)	1	63 (34.4)	37 (46.8)	0.44
EQ-5D-5L	*n* = 77	*n* = 46		*n* = 153	*n* = 109	
1. I have no pain or discomfort	37 (48.1)	21 (45.7)		51 (33.3)	27 (24.8)	
2. I have slight pain or discomfort	38 (49.4)	22 (47.8)	0.85	86 (56.2)	60 (55.0)	0.17
3. I have moderate pain or discomfort	2 (2.6)	3 (6.5)		16 (10.5)	19 (17.4)	
4. I have severe pain or discomfort	0 (0)	0 (0)		0 (0)	3 (2.8)	

ASDAS = Ankylosing Spondylitis Disease Activity Score; ASQoL = Ankylosing Spondylitis Quality; EQ-5D-5L = EuroQoL 5-Dimension 5-Level. Data are presented as *n* (%).

**Table 7:** Quality of life in patients with axial spondyloarthritis using ASQoL and EQ-5D-5L stratified by disease duration and ASDAS.

Variable	≤7 Years	>7 Years		≤7 Years	>7 Years	
	<1.3 ASDAS	<1.3 ASDAS	*p-*Value	<2.1 ASDAS	<2.1 ASDAS	*p*-Value
ASQoL	*n* = 50	*n* = 73		*n* = 126	*n* = 136	
Q7: I feel tired all day	3 (6.0)	9 (12.3)	0.36	29 (23.0)	26 (19.1)	0.55
Q8: I have to stop what I am doing to rest	3 (6.0)	8 (11.0)	0.52	25 (19.8)	26 (19.1)	1.00
Q12: I get tired easily	11 (22.0)	19 (26.0)	0.67	47 (37.6)	53 (39.3)	0.80
EQ-5D-5L	*n* = 50	*n* = 73		*n* = 126	*n* = 136	
1. I have no pain or discomfort	25 (52.0)	32 (43.8)		38 (30.2)	40 (29.4)	
2. I have slight pain or discomfort	23 (46.0)	37 (50.7)	0.46	68 (54)	78 (57.4)	1.00
3. I have moderate pain or discomfort	1 (2.0)	4 (5.5)		19 (15.1)	16 (11.8)	
4. I have severe pain or discomfort	0 (0)	0 (0)		1 (0.8)	2 (1.5)	

ASDAS = Ankylosing Spondylitis Disease Activity Score; ASQoL = Ankylosing Spondylitis Quality; EQ-5D-5L = EuroQoL 5-Dimension 5-Level. Data are presented as *n* (%).

## Data Availability

Data are available upon reasonable request. All data relevant to the study are included in the article. The datasets generated or analysed during the current study are not publicly available. AbbVie is committed to sharing with qualified external researchers’ access to patient-level data and supporting clinical documents from eligible studies. All data used in this study were anonymised to respect the privacy of patients in line with applicable laws and regulations.
